# A cross-sectional MR study of body fat volumes and distribution in chronic schizophrenia

**DOI:** 10.1038/s41537-022-00233-z

**Published:** 2022-03-18

**Authors:** Emanuele F. Osimo, Stefan P. Brugger, E. Louise Thomas, Oliver D. Howes

**Affiliations:** 1 MRC London Institute of Medical Sciences, Institute of Clinical Sciences, Faculty of Medicine, Imperial College London, Hammersmith Hospital Campus, London, UK; 2 Department of Psychiatry, University of Cambridge, Cambridge, UK; 3 Cambridgeshire and Peterborough NHS Foundation Trust, Cambridge, UK; 4 South London and Maudsley NHS Foundation Trust, London, UK; 5 Cardiff University Brain Research and Imaging Centre, School of Psychology, Cardiff University, London, UK; 6 Research Centre for Optimal Health, School of Life Sciences, University of Westminster, London, UK; 7 Institute of Psychiatry, Psychology and Neuroscience, King’s College London, London, UK

**Keywords:** Molecular neuroscience, Schizophrenia

## Abstract

People with schizophrenia show higher risk for abdominal obesity than the general population, which could contribute to excess mortality. However, it is unclear whether this is driven by alterations in abdominal fat partitioning. Here, we test the hypothesis that individuals with schizophrenia show a higher proportion of visceral to total body fat measured using magnetic resonance imaging (MRI). We recruited 38 participants with schizophrenia and 38 healthy controls matched on age, sex, ethnicity, and body mass index. We found no significant differences in body fat distribution between groups, suggesting that increased abdominal obesity in schizophrenia is not associated with altered fat distribution.

People with schizophrenia show a 10–15 years shorter life expectancy^1^, and their mortality due to natural causes is up to 8-times higher than expected^2^. They have been shown to have an increased risk for abdominal obesity compared to the general population (odds ratio of 4.4 in a meta-analysis^3^). Abdominal obesity is the most prominent feature of the metabolic syndrome, and associates with blood lipid disorders, inflammation, insulin resistance or diabetes, and, downstream, increased risk of developing cardiovascular disease^4^. Obesity, therefore, might underpin many of the metabolic comorbidities that are seen in schizophrenia, including a higher prevalence of hypertension, high cholesterol and triglycerides, type 2 diabetes, insulin resistance, and the metabolic syndrome^3^. These, in turn, might be responsible for the increased cardiovascular mortality in this group^5^.

Few cross-sectional studies have investigated visceral adipose tissue mass or volume in treated schizophrenia^6^. Some have used proxies, such as bioelectrical impedance (BIA)^7,8^, while others have used gold standard magnetic resonance imaging (MRI)^9,10^ to visualise body fat distributions in cases and controls with conflicting results. MRI studies have reported no significant difference in visceral fat content between cases and controls^9,10^, whereas studies using BIA have shown both increases^7^ or decreases^8^ in visceral fat in schizophrenia. However, studies measuring visceral fat using MRI tend not to measure total body fat content, and it is unclear whether these studies are able to detect differences in distribution at the whole-body level in schizophrenia.

Our hypothesis was that participants with schizophrenia and controls, matched for body mass index (BMI), would show similar levels of total body fat, but an increased visceral fraction, as compared to controls.

Thirty-eight participants with schizophrenia, as well as the same number of matched healthy controls, underwent whole body fat MRI measures. As expected, due to the nature of case control matching there were no significant group differences for age, sex, ethnicity and BMI. A sample description is in Supplementary Table 1.

Table 1 describes MRI-derived fat measures according to diagnostic status. There were no significant differences between groups in total fat (*d* = 0.00, 95% CI: −0.46–0.46; *p* = 1.00), visceral fat (*d* = 0.06, 95% CI: −0.40–0.52; *p* = 0.79), or visceral to total ratio (*d* = 0.17, 95% CI: −0.29–0.62; *p* = 0.47).

**Table 1:** MRI-derived measurements in participants with schizophrenia and matched healthy controls.

Characteristic	Sample size (*N* schizophrenia, *N* HC)	Schizophrenia (Mean (SD))	Healthy controls (Mean (SD))	Statistical results	Effect size (Cohen’s *d*; 95% CI)
Total body fat (L)	38, 38	30.27 (15.9)	30.29 (15.2)	*F* (1, 74) = 0.00, *p* = 1.0	0.00; −0.46–0.46
Visceral fat (L)	38, 38	3.83 (1.86)	3.72 (1.92)	*F* (1, 74) = 0.07, *p* = 0.79	0.06; −0.40–0.52
Visceral fat ratio Visceral/total body fat	38, 38	0.13 (0.04)	0.13 (0.05)	*F* (1, 74) = 0.52, *p* = 0.47	0.17; −0.29–0.62

*HC* healthy controls, *SD* standard deviation, *CI* confidence interval, *p* = *p* value.

In this work, we investigate the body fat distribution of people with chronic schizophrenia compared to healthy controls matched for age, sex, ethnicity, and BMI.

We show that subjects with schizophrenia do not show any differences in overall adipose tissue content or regional distribution, when compared to matched healthy controls. This compares to the presence of concentric cardiac remodelling and cardiac fibrosis in the same patient cohort^11,12^.

Our results are consistent with the majority of prior cross-sectional studies of visceral body fat in treated schizophrenia using a comparable methodology, and extend the previous literature on the topic by using whole-body MR imaging, to show that both body fat content and distribution were not different between cases vs. controls^9,10,13–15^. A recent meta-analysis^6^ compared levels of visceral adipose fat between people with schizophrenia and controls, showing significant increases in the treated sample (*N* = 53)—however the team could only include in the quantitative synthesis three^13–15^ out of the eight existing studies^7–10,13–16^. In Supplementary Table 2, we present our own summary of existing studies, showing that studies showing differences in visceral body fat mass between treated patients with schizophrenia and controls either did not match cases and controls by BMI, or used indirect measurement techniques, such as bioimpedance analysis.

Crucial to the interpretation of our work is that all participants with schizophrenia we included were chronic patients taking second-generation antipsychotics (SGAs), mostly clozapine and olanzapine. SGAs are known to cause increases in body weight^17^, and are potentially one of the causes of the increased prevalence of obesity in schizophrenia. Therefore, the finding of no difference in fat distribution in this sample, despite participants being exposed to these metabolically active compounds, is particularly meaningful. On the flip side, the findings in this cohort might not extend to untreated or minimally treated cohorts of patients with schizophrenia, so further research is needed in untreated patients. However, we note that previous studies looking at abdominal fat mass differences between drug-naïve people with a first episode of psychosis and matched healthy controls found no difference even before antipsychotic drugs were started—as evidenced in a recent meta-analysis^6^ after removing poor quality studies.

In this work, we find no differences in total or visceral fat volumes between treated people with schizophrenia and matched healthy controls. This does not imply no difference exists in terms of adipose tissue function in schizophrenia, as this cannot be captured by MRI. Indeed, previous research has shown that antipsychotics can cause a pro-inflammatory shift in adipose tissue in mice^18^; in humans, we have previously found that despite no differences in body-fat distribution, people with antipsychotic-treated chronic schizophrenia are characterised by alterations in systemic adipokine levels and pro-inflammatory changes^19^. Collectively, finding no differences in visceral body fat mass/distribution, while at the same time finding signs of adipocyte dysfunction and pro-inflammatory changes, might suggest that chronic, treated schizophrenia is characterised by the activation of specific molecular pathways^19^. These pathways might lead downstream to cardio-metabolic changes that might explain part of the additional cardiovascular morbidity and mortality seen in schizophrenia^3^.

Strengths of our study include excluding any history of cardiometabolic disease, including diabetes, hypertension, dyslipidaemia, and ischaemic heart disease, to make the case and control groups more comparable, given the different prevalence of these conditions in the two cohorts. Future larger studies could include and adjust the analyses for the presence of these conditions. In terms of limitations, it is possible that our negative findings might be down to a statistical type II error, however our sample size was among the largest of similar studies, and our effect size measures were very close to 0 for all outcomes. Further, we were able to match our samples for age, sex, ethnicity, and BMI. Data about further confounds, such as smoking, activity levels, and diet were not available for part of the cohort and could not be presented.

The lack of differences in visceral fat we observed in a closely matched control–case study add strength to similar findings from previous MRI studies. Differences in the prevalence of cardiometabolic disease in schizophrenia cannot be explained by specific alterations in body fat partitioning. Prospective studies will be useful to determine whether disease-related biological factors play a specific role in cardiometabolic disease in schizophrenia, or whether they simply reflect the same disease processes found in the wider population.

## Methods

### Participants

People with schizophrenia were recruited from community mental health services in London, UK. Healthy controls were recruited through the Hammersmith Hospital Healthy Volunteer Panel, London, UK, and through direct advertising, and were matched to patients for age (+/− 3 years), ethnicity, sex, and BMI (+/− 1).

Exclusion criteria for all participants were: age <18 or >65 years, pregnancy or breastfeeding, a history of cardiometabolic disease, including diabetes, hypertension, dyslipidaemia, ischaemic heart disease, any vascular disorder, other history of congenital/structural cardiac disease; or history of significant or continuing substance abuse. Inclusion criterion for patients was an ICD-10 diagnosis of schizophrenia. Exclusion criterion for healthy controls was a previous history or first-degree family history of schizophrenia or other psychotic disorder.

Written informed consent was obtained from all volunteers. The authors assert that all procedures contributing to this work comply with the ethical standards of the relevant national and institutional committees on human experimentation and with the Helsinki Declaration of 1975, as revised in 2008. All procedures involving human subjects were approved by the London—Camberwell St Giles Research Ethics Committee.

In addition to reviewing medical records, all subjects received a medical review and clinical examination to exclude medical co-morbidities. BMI was calculated as body mass (kg) divided by the square of body height (m^2^).

The patient sample in this study overlaps with our previously published MRI studies including cardiac and body fat measures by MRI^11,12,19^, while healthy controls show only partial overlap.

### Magnetic resonance imaging

MR imaging was performed at a single site for all participants. All participants but 29 healthy controls were scanned on a 3 T Siemens Magnetom Prisma (Erlangen, Germany) using a combination of a 18-channel body coil and 12 elements of a 32-channel spine coil. An additional 29 whole-body fat imaging datasets were obtained at the same scanning site from healthy controls using a 1.5 T Phillips Achiva scanner (Phillips, Best, the Netherlands). Previous QA measures comparing whole-body fat imaging datasets from individuals scanned on both 1.5 and 3 T scanners demonstrate the data could be combined. A study of within-scanner variability (reproducibility) and across-scanner variability (including both 3 and 1.5 T scanners) found that the within-scanner repeatability (coefficient of variation = 2.9%) explained much of the overall reproducibility (CoV = 4.4%) for visceral fat volumes^20^.

Fat content and distribution were determined as follows: subjects were scanned using a rapid whole-body T1-weighted MRI protocol. Images were analysed by operators blind to diagnosis using SliceOmatic (Tomovision, Montreal, Quebec, Canada) and regional volumes were recorded in litres (L), including total adipose tissue and visceral fat^21,22^.

### Statistical analysis

Differences between patients and controls were tested using *χ*^2^ tests for categorical variables, Kruskal–Wallis tests by ranks for non-normally distributed values, and analysis of variance (anova) for normally distributed measures. Effect sizes were calculated using Cohen’s *d* measure.

In all tests, a *p* value <0.05 (two-tailed) was taken as significant. Statistical analyses were performed in R.

### Reporting summary

Further information on research design is available in the Nature Research Reporting Summary linked to this article.

## Supplementary information

Supplementary tables

Reporting Summary

## Data Availability

The data that support the findings of this study are available on request from the corresponding authors. The data are not publicly available due to ethics constraints and the potential for breaching participant privacy.

## References

[1] Plana-Ripoll O (2019). A comprehensive analysis of mortality-related health metrics associated with mental disorders: a nationwide, register-based cohort study. Lancet.

[2] Harris C, Barraclough B (1998). Excess mortality of mental disorder. Br. J. Psychiatry.

[3] Vancampfort D (2013). A meta‐analysis of cardio‐metabolic abnormalities in drug naïve, first‐episode and multi‐episode patients with schizophrenia versus general population controls. World Psychiatry.

[4] Després J-P, Lemieux I (2006). Abdominal obesity and metabolic syndrome. Nature.

[5] Laursen TM, Munk-Olsen T, Vestergaard M (2012). Life expectancy and cardiovascular mortality in persons with schizophrenia. Curr. Opin. Psychiatry.

[6] Smith E (2021). Adiposity in schizophrenia: a systematic review and meta‐analysis. Acta Psychiatr. Scand..

[7] Konarzewska B (2014). Visceral obesity in normal-weight patients suffering from chronic schizophrenia. BMC Psychiatry.

[8] Kornetova EG (2020). Body fat parameters, glucose and lipid profiles, and thyroid hormone levels in schizophrenia patients with or without metabolic syndrome. Diagnostics.

[9] Kim J-H (2017). Body and liver fat content and adipokines in schizophrenia: a magnetic resonance imaging and spectroscopy study. Psychopharmacology.

[10] Ruppert J (2018). Increased pericardial adipose tissue and cardiometabolic risk in patients with schizophrenia versus healthy controls. Eur. Arch. Psychiatry Clin. Neurosci..

[11] Osimo EF (2020). Cardiac structure and function in schizophrenia: a cardiac MR imaging study. Br. J. Psychiatry.

[12] Pillinger T (2019). Cardiac structure and function in patients with schizophrenia taking antipsychotic drugs: an MRI study. Transl. Psychiatry.

[13] Chouinard V-A (2019). Impaired insulin signaling in unaffected siblings and patients with first-episode psychosis. Mol. Psychiatry.

[14] Kozłowska E (2019). The expression of toll-like receptors in peripheral blood mononuclear cells is altered in schizophrenia. Psychiatry Res..

[15] Sapra M, Lawson D, Iranmanesh A, Varma A (2016). Adiposity-independent hypoadiponectinemia as a potential marker of insulin resistance and inflammation in schizophrenia patients treated with second generation antipsychotics. Schizophr. Res..

[16] Blouin M (2008). Adiposity and eating behaviors in patients under second generation antipsychotics. Obesity.

[17] Pillinger T (2020). Comparative effects of 18 antipsychotics on metabolic function in patients with schizophrenia, predictors of metabolic dysregulation, and association with psychopathology: a systematic review and network meta-analysis. Lancet Psychiatry.

[18] Calevro A (2018). Effects of chronic antipsychotic drug exposure on the expression of translocator protein and inflammatory markers in rat adipose tissue. Psychoneuroendocrinology.

[19] Osimo EF (2021). Adipose tissue dysfunction, inflammation, and insulin resistance: alternative pathways to cardiac remodelling in schizophrenia. A multimodal, case–control study. Transl. Psychiatry.

[20] Borga M (2020). Reproducibility and repeatability of MRI‐based body composition analysis. Magn. Reson. Med..

[21] O’donovan G (2009). Fat distribution in men of different waist girth, fitness level and exercise habit. Int. J. Obes..

[22] Thomas EL (2012). The missing risk: MRI and MRS phenotyping of abdominal adiposity and ectopic fat. Obesity.

