# Supplementary material for: A cross-sectional MR study of body fat volumes and distribution in chronic schizophrenia
Source: Schizophrenia (Heidelb). 2022 Mar 18;8(1):24. doi: 10.1038/s41537-022-00233-z (PMC8933542; doi:10.1038/s41537-022-00233-z)
Supplement: Supplementary file 1 — Supplementary tables [file 41537_2022_233_MOESM1_ESM.pdf]

Supplementary Tables

Supplementary Table 1: Sample Characteristics

Characteristic	Schizophrenia	Healthy Controls	Test statistic and p value
Sample size	38	38	
Male sex, N (%)	29 (76%)	29 (76%)	χ^2 = 0; df=1; p=1
White ethnicity, N (%)	17 (47%)	19 (50%)	χ^2 = 0.1; df=1; p=0.78
Age, years mean (SD)	40.2 (10.0)	39.7 (10.2)	t = 0.2, df = 74, p = 0.84
BMI (kg/m2), mean (SD)	28.45 (6.07)	28.53 (5.44)	t = -0.06, df = 73, p = 0.95
Chlorpromazine Equivalent Dose (mg/day), median (IQR)	359 (274)	0 (NA)	NA
Duration of treatment (years), median (IQR)	12 (12.5)	NA	NA

df: degrees of freedom; p= p value; χ^2: chi squared; SD: standard deviation; IQR: inter-quartile range; NA: not available; BMI: body mass index.

Supplementary Table 2: Comparison of existing cross-sectional studies of visceral body fat in treated schizophrenia

Study	Case vs controls Visceral fat	BMI-matched	Patient N	Method for assessing visceral fat	Patients' treatment
Blouin et al, 2008	Significantly increased	No	18	computed tomography	SGA
Chouinard et al, 2019	No difference	Yes	18	whole-body dual-energy X-ray absorptiometry (DXA)	Half on antipsychotics
Kim et al, 2017	No difference	Yes	13	MR	SGA
Konarzewska et al, 2014	Significantly increased	Yes	52	bioelectrical impedance analysis (BIA)	Atypical or typical antipsychotic agents
Kornetova et al, 2020	Significantly reduced	No	156	bioimpedance analysis on scale	Treated
Kozlowska et al, 2019	No difference	Yes	27	dual-energy X-ray absorptiometry (DXA)	Most on antipsychotic polytherapy
Ruppert et al, 2018	No difference	No	31	MR	Treated
Sapra et al, 2016	No difference	Yes	8	dual-energy X-ray absorptiometry (DXA)	SGA

SGA: second generation antipsychotics; N: sample size; BMI: body mass index; MR: magnetic resonance
