# Supplementary material for: A cross-sectional MR study of body fat volumes and distribution in chronic schizophrenia
Source: Schizophrenia (Heidelb). 2022 Mar 18;8(1):24. doi: 10.1038/s41537-022-00233-z (PMC8933542; doi:10.1038/s41537-022-00233-z)
Supplement: Supplementary file 2 — Reporting Summary [file 41537_2022_233_MOESM2_ESM.pdf]

nature portfolio

Corresponding author(s): Dr Emanuele F Osimo
Last updated by author(s): 13/12/2021

Reporting Summary

Nature Portfolio wishes to improve the reproducibility of the work that we publish. This form provides structure for consistency and transparency in reporting. For further information on Nature Portfolio policies, see our Editorial Policies and the Editorial Policy Checklist.

Statistics

For all statistical analyses, confirm that the following items are present in the figure legend, table legend, main text, or Methods section.

n/a	Confirmed	
☐	☒	The exact sample size (n) for each experimental group/condition, given as a discrete number and unit of measurement
☐	☒	A statement on whether measurements were taken from distinct samples or whether the same sample was measured repeatedly
☐	☒	The statistical test(s) used AND whether they are one- or two-sided<br>*Only common tests should be described solely by name; describe more complex techniques in the Methods section.*
☐	☒	A description of all covariates tested
☐	☒	A description of any assumptions or corrections, such as tests of normality and adjustment for multiple comparisons
☐	☒	A full description of the statistical parameters including central tendency (e.g. means) or other basic estimates (e.g. regression coefficient) AND variation (e.g. standard deviation) or associated estimates of uncertainty (e.g. confidence intervals)
☐	☒	For null hypothesis testing, the test statistic (e.g. F, t, r) with confidence intervals, effect sizes, degrees of freedom and P value noted<br>*Give P values as exact values whenever suitable.*
☒	☐	For Bayesian analysis, information on the choice of priors and Markov chain Monte Carlo settings
☒	☐	For hierarchical and complex designs, identification of the appropriate level for tests and full reporting of outcomes
☐	☒	Estimates of effect sizes (e.g. Cohen's d, Pearson's r), indicating how they were calculated

Our web collection on statistics for biologists contains articles on many of the points above.

Software and code

Policy information about availability of computer code

Data collection	Data collected in spreadsheets.
Data analysis	Statistical analyses were performed in R.

For manuscripts utilizing custom algorithms or software that are central to the research but not yet described in published literature, software must be made available to editors and reviewers. We strongly encourage code deposition in a community repository (e.g. GitHub). See the Nature Portfolio guidelines for submitting code & software for further information.

Data

Policy information about availability of data

All manuscripts must include a data availability statement. This statement should provide the following information, where applicable:

- Accession codes, unique identifiers, or web links for publicly available datasets
- A description of any restrictions on data availability
- For clinical datasets or third party data, please ensure that the statement adheres to our policy

The data that support the findings of this study are available on request from the corresponding authors. The data are not publicly available due to ethics constraints and the potential for breaching participant privacy.

Field-specific reporting

Please select the one below that is the best fit for your research. If you are not sure, read the appropriate sections before making your selection.

☒ Life sciences ☐ Behavioural & social sciences ☐ Ecological, evolutionary & environmental sciences

For a reference copy of the document with all sections, see nature.com/documents/nr-reporting-summary-flat.pdf

Life sciences study design

All studies must disclose on these points even when the disclosure is negative.

Sample size	No specific sample size calculation was performed. However, our patient sample size of 38 patients and 38 controls is considered to be powered to detect a difference in visceral and total body fat, given that all previous studies of fat content in schizophrenia using the same MR technique were smaller (the biggest included 31 patients).
Data exclusions	No data were excluded from the analysis.
Replication	As discussed in the paper, our results are consistent with the majority of prior cross-sectional studies of visceral body fat in treated schizophrenia using a comparable methodology, and extend the previous literature on the topic by using whole-body MR imaging, to show that both body fat content and distribution were not different between cases vs controls
Randomization	This was a case-control study, and therefore no randomisation took place. Controls were matched to patients for age (+/- 3 years), ethnicity, sex, and BMI (+/- 1).
Blinding	MR operators and image analysis took place blind to diagnosis.

Reporting for specific materials, systems and methods

We require information from authors about some types of materials, experimental systems and methods used in many studies. Here, indicate whether each material, system or method listed is relevant to your study. If you are not sure if a list item applies to your research, read the appropriate section before selecting a response.

Materials & experimental systems

n/a	Involved in the study	
☒	☐	Antibodies
☒	☐	Eukaryotic cell lines
☒	☐	Palaeontology and archaeology
☒	☐	Animals and other organisms
☐	☒	Human research participants
☐	☒	Clinical data
☒	☐	Dual use research of concern

Methods

n/a	Involved in the study	
☒	☐	ChIP-seq
☒	☐	Flow cytometry
☒	☐	MRI-based neuroimaging

Human research participants

Policy information about studies involving human research participants

Population characteristics	Please see supplementary table 1
Recruitment	People with schizophrenia were recruited from community mental health services in London, UK. Healthy controls were recruited through the Hammersmith Hospital Healthy Volunteer Panel, London, UK, and through direct advertising, and were matched to patients for age (+/- 3 years), ethnicity, sex, and BMI (+/- 1).<br>Exclusion criteria for all participants were: age <18 or >65 years, pregnancy or breastfeeding, a history of cardiometabolic disease, including diabetes, hypertension, dyslipidaemia, ischaemic heart disease, any vascular disorder, other history of congenital/structural cardiac disease; or history of significant or continuing substance abuse. Inclusion criterion for patients was an ICD-10 diagnosis of schizophrenia. Exclusion criterion for healthy controls was a previous history or first-degree family history of schizophrenia or other psychotic disorder.
Ethics oversight	Written informed consent was obtained from all volunteers. The authors assert that all procedures contributing to this work comply with the ethical standards of the relevant national and institutional committees on human experimentation and with the Helsinki Declaration of 1975, as revised in 2008. All procedures involving human subjects were approved by the London - Camberwell St Giles Research Ethics Committee.

Note that full information on the approval of the study protocol must also be provided in the manuscript.

Clinical data

Policy information about clinical studies
All manuscripts should comply with the ICMJE guidelines for publication of clinical research and a completed CONSORT checklist must be included with all submissions.

Clinical trial registration	N/A as this is a non-CTIMP
Study protocol	N/A
Data collection	Data was collected at Imperial College London between September 2015 and December 2018.
Outcomes	Our hypothesis was that participants with schizophrenia and controls, matched for body mass index (BMI), would show similar levels of total body fat, but an increased visceral fraction, as compared to controls.
